# Development of a new caged intein for multi-input conditional translation of synthetic mRNA

**DOI:** 10.1038/s41598-024-60809-w

**Published:** 2024-05-01

**Authors:** Tingting Yang, Hideyuki Nakanishi, Keiji Itaka

**Affiliations:** 1 Department of Biofunction Research, Institute of Biomaterials and Bioengineering, Tokyo Medical and Dental University (TMDU), Tokyo, 101-0062 Japan; 2 Center for Infectious Disease Education and Research (CiDER), Osaka University, Osaka, 565-0871 Japan

**Keywords:** Biotechnology, Gene therapy, Nucleic-acid therapeutics, Genetic circuit engineering, Logic gates, Protein engineering, Synthetic biology

## Abstract

mRNA medicines can be used to express therapeutic proteins, but the production of such proteins in non-target cells has a risk of adverse effects. To accurately distinguish between therapeutic target and nontarget cells, it is desirable to utilize multiple proteins expressed in each cell as indicators. To achieve such multi-input translational regulation of mRNA medicines, in this study, we engineered *Rhodothermus marinus* (Rma) DnaB intein to develop “caged Rma DnaB intein” that enables conditional reconstitution of full-length translational regulator protein from split fragments. By combining the caged Rma DnaB intein, the split translational regulator protein, and target protein-binding domains, we succeeded in target protein-dependent translational repression of mRNA in human cells. In addition, the caged Rma intein showed orthogonality to the previously reported *Nostoc punctiforme* (Npu) DnaE-based caged intein. Finally, by combining these two orthogonal caged inteins, we developed an mRNA-based logic gate that regulates translation based on the expression of multiple intracellular proteins. This study provides important information to develop safer mRNA medicines.

## Introduction

Messenger RNAs (mRNAs) are single-stranded RNAs that have a pivotal role in gene expression, where the information of a gene is used to produce proteins. In gene expression, mRNA is generated through transcription from DNA, and a series of modifications such as capping at the 5′ end. The mature mRNA is sent out from the nucleus into the cytoplasm, where the ribosome binds and begins to translate the mRNA into the protein.

mRNA medicines, which are artificially synthesized by in vitro transcription from template DNAs, are considered to produce proteins based on a similar innate mechanism. Consequently, any cell can produce the encoded proteins from the administered mRNAs. However, therapeutic gene expression in non-target organs or cells may occasionally cause adverse effects^1–3^. If the mRNAs can have the capacity of cell-specific protein translation, the mRNA medicines can be safer and more target-directed.

In order to control the translation of mRNA medicines according to cell type and conditions, in a previous study^4^, our group developed an intracellular protein-responsive translational regulation system to achieve cell-specific protein translation from the administered mRNAs. An important part of this system is the Caliciviral VPg-based translational activator (CaVT). CaVT was obtained by fusing a dlFG mutant^5^ of the bacteriophage MS2 coat protein (MS2CP) with the feline caliciviral VPg protein^6–8^. MS2CP is a motif-specific RNA-binding protein that is widely used in RNA-based mammalian gene circuits^7–13^. Caliciviral VPg is a 5′ cap mimetic protein that interacts with the eukaryotic translation initiation factor 4F (eIF4F) complex^14^. Importantly, CaVT can perform both translational repression and activation of synthetic mRNAs using an affinity-dependent manner^4^. When the target mRNA contains a strong MS2 binding motif, CaVT inhibits the translation of the target mRNA by MS2CP^6–8^. In contrast, when the target mRNA lacks a canonical 5′-cap structure but contains a weak MS2 binding motif, CaVT can activate the translation of the target mRNA by caliciviral VPg.

In a previous study, to develop protein-responsive CaVT that can be used for conditional translational activation and repression, we combined CaVT split within MS2CP and an engineered protein called “caged intein”^4^. Inteins are protein domains that excise themselves from their precursor proteins. An intein is flanked by protein domains called exteins, and these two exteins are ligated by a peptide bond when the intein is excised from its precursor^15^. This post-translational excision and ligation process is called “protein splicing”. Some inteins consist of two separately translated proteins, N- and C- inteins, and are called “split intein”. In the case of split inteins, N- and C-inteins spontaneously associate with each other, followed by intein-excision and extein-ligation processes like contiguous inteins. The protein splicing of split inteins, which is called "protein trans-splicing", has been utilized to ligate two separately translated proteins, but conventional protein trans-splicing is unconditional. For the purpose of regulating protein trans-splicing, Gramespacher et al. developed a caged intein based on *Nostoc punctiforme* (Npu) DnaE^16,20^ and achieved conditional protein splicing. The caged intein is expressed as two fragments called caged N- and caged C-inteins and induces protein splicing only when caged N- and caged C-inteins are very close. In their study, the caged N-intein was developed by adding the N-terminal fragment of C-intein to N-intein. Similarly, to develop caged C-intein, they added the C-terminal fragment of N-intein to C-intein.

Using the caged Npu DnaE intein fused with split fragments of CaVT and antibody-derived target protein-binding domains called “nanobody”, we achieved target protein-dependent reconstitution of full-length CaVT by conditional protein splicing^4^. This protein-responsive translational regulation system provides a possibility to achieve selective translation in target protein-expressing cells to make mRNA medicine more functional and safer. However, due to the limited variety of caged intein, simultaneous reconstitution of multiple translational regulator proteins is difficult. This limitation makes multi-input translational regulation difficult.

Therefore, in this study, we developed a new caged intein based on thermophilic eubacterium *Rhodothermus marinus* (Rma) DnaB intein, whose protein splicing efficiency in mammalian cells is very high^15^. Similar to the caged Npu DnaE intein, the caged Rma DnaB intein enabled protein-responsive translational regulation. Furthermore, the caged Rma DnaB intein showed orthogonality to the caged Npu DnaE intein, which allows the usage of both caged inteins in the identical translational regulation system. Finally, we constructed the logic gate by integrating the caged Rma DnaB intein and the caged Npu DnaE intein in the identical system and achieved translational regulation using two intracellular proteins as inputs.

## Results

### Comparison of normal and C-terminally truncated Rma N-inteins

**Figure 1:**
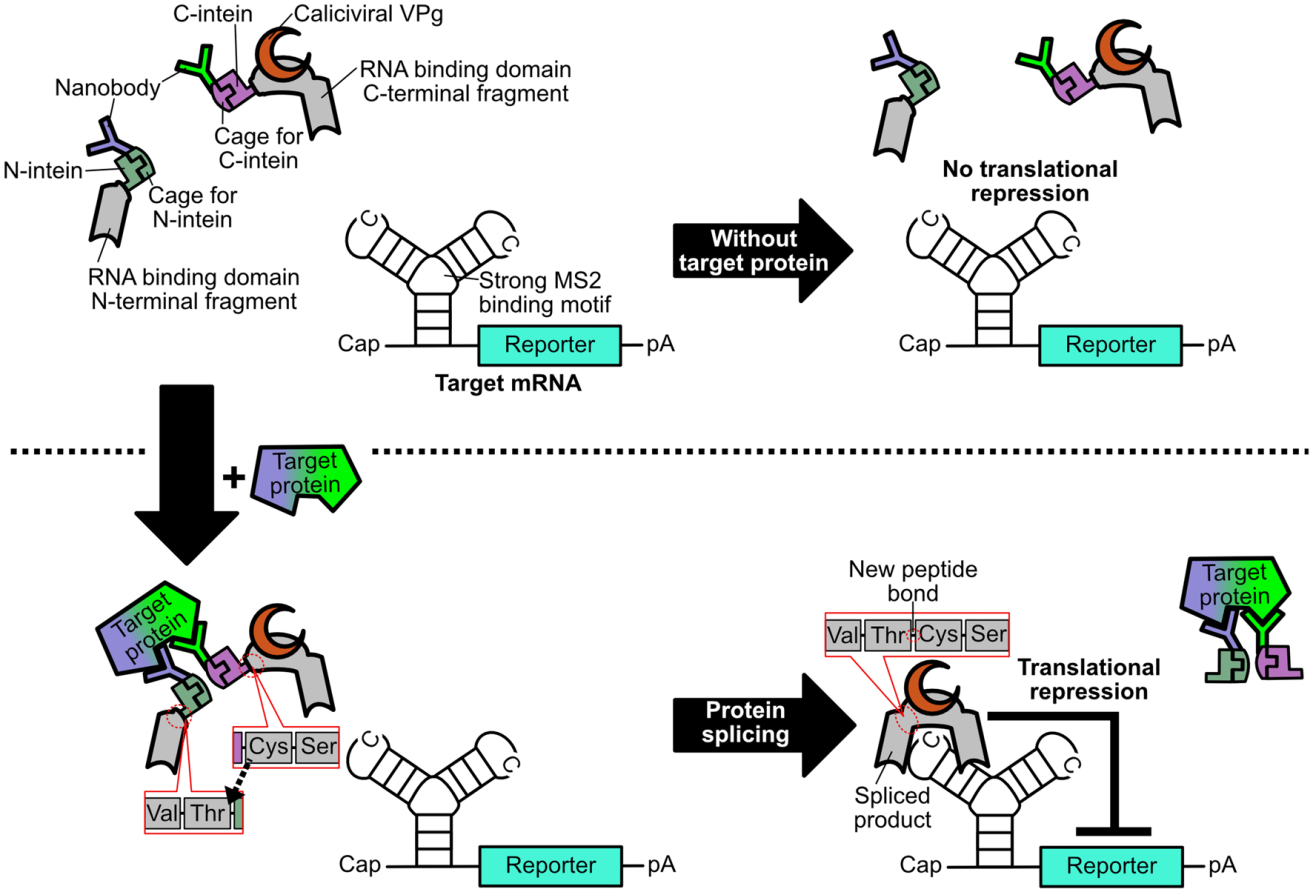
Mechanism of protein-responsive translational regulation.

Figure 1 shows a strategy for target protein-responsive translational repression. In the absence of the target protein, the nanobody does not direct the split CaVT protein in close proximity to each other and thus does not induce protein splicing. Consequently, the full-length CaVT is not reconstituted, and the translation of the target mRNA is not repressed. Conversely, in the presence of the target protein, the binding of the nanobody to the target protein brings the split CaVT proteins in close proximity to each other and reconstitutes the full-length CaVT through protein splicing of the caged intein. Then, the reconstituted full-length CaVT binds to the MS2 binding motif in the target mRNA through MS2CP and represses the translation of the target mRNA.

In order to establish a multi-input translational regulation system, the first step is to develop a caged intein that is orthogonal to the previously used caged Npu DnaE intein. Here, we used the Rma DnaB intein as the basis for the design of caged inteins. Because previous studies reported that the N-terminal 106 amino acids (aa) and the C-terminal 51 aa of Rma DnaB intein can be used as N- and C-inteins respectively^17,18^, we used these regions as the basis for the caged intein design. First, to check the protein trans-splicing efficiency of the split Rma DnaB intein, we used the cage-free N- and C-inteins which can induce spontaneous protein splicing regardless of the target protein. We fused N- and C-inteins to N- and C-terminal fragments of the C46-split CaVT (the CaVT split at the cysteine residue at position 46) respectively, because the C46-split CaVT showed translational repression only when it is reconstituted to the full-length CaVT^4^.

**Figure 2:**
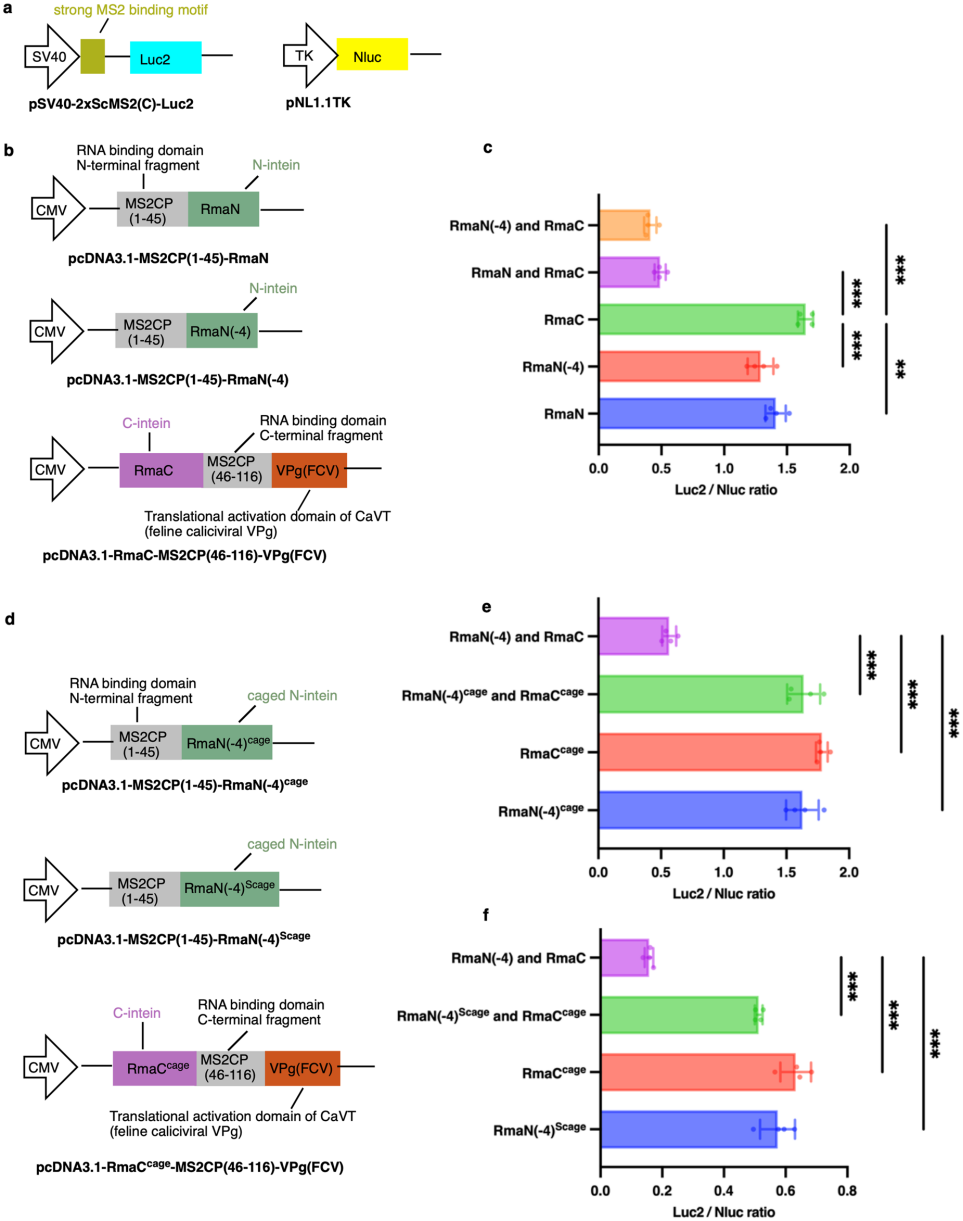
Translational repression by the reconstituted CaVT in pDNA transfection. (**a**) Reporter pDNAs to evaluate translational repression. While the firefly luciferase (Luc2) expression vector called pSV40-2xScMS2(C)-Luc2 contains the target motif for translational repression, the Nluc expression vector called pNL1.1TK[Nluc/TK] lacks the motif and was used as a control. (**b**) pDNAs of split CaVT with uncaged inteins to check translational repression induced by spontaneous reconstitution. (**c**) HeLa cells were co-transfected with pSV40-2xScMS2(C)-Luc2 (40 ng/well), pNL1.1TK[Nluc/TK] (20 ng/well), and the pDNAs to express split CaVT (total 40 ng/well). Cells transfected with only one of pcDNA3.1-MS2CP(1–45)-RmaN, pcDNA3.1-MS2CP(1–45)-RmaN(-4), or pcDNA3.1-RmaC-MS2CP(46–116)-VPg(FCV) were used as negative controls for translational repression. The bar graph shows the Luc2/Nluc ratio (mean ± SD, n = 4). **P < 0.01, ***P < 0.001 compared to one of the negative controls (pcDNA3.1-RmaC-MS2CP(46–116)-VPg(FCV)) by Tukey’s multiple comparison test. (**d**) pDNAs to check the inhibitory effect of designed cages on unconditional reconstitution of split CaVT. (**e**–**f**) HeLa cells were co-transfected with pSV40-2xScMS2(C)-Luc2 (40 ng/well), pNL1.1TK[Nluc/TK] (20 ng/well), and the pDNAs to express the caged split CaVT (total 40 ng/well). The positive control for translational repression is pcDNA3.1-MS2CP(1–45)-RmaN(-4) and pcDNA3.1-RmaC-MS2CP(46–116)-VPg(FCV) group. For the N-terminal fragment of C46-split CaVT, two types of caged RmaN(-4), named RmaN(-4)^cage^ (**e**) and Rma(-4)^Scage^ (**f**) respectively, were tested. The bar graph shows the Luc2/Nluc ratio (mean ± SD, n = 4). ***P < 0.001 compared with the positive control by Tukey’s multiple comparison test.

For this purpose, we designed three vectors. One is the vector to express the N-terminal fragment of C46-split CaVT fused with the normal Rma N-intein (RmaN). The second is the N-terminal fragment of C46-split CaVT fused with the variant of Rma N-intein with the C-terminal 4 amino acid residues removed (RmaN(-4)) whose high protein splicing efficiency was previously reported^19^. The third one is the C-terminal fragment of C46-split CaVT fused with the Rma C-intein (RmaC). To compare the protein splicing efficiency, the pDNA expressing C46-split CaVT was co-transfected with a firefly luciferase (Luc2) expression vector called pSV40-2xScMS2(C)-Luc2 which containing a strong MS2 binding motif. We also co-transfected control reporter pDNA called pNL1.1TK[Nluc/TK] into HeLa cells to express *Oplophorus gracilirostris*-derived NanoLuc (Nluc) as a transfection control (Fig. 2a,b). As shown in Fig. 2c, the combination of RmaN(-4) and RmaC caused slightly stronger translational repression than that of RmaN and RmaC. This result suggests a slightly higher protein splicing efficiency of RmaN(-4). So, we used RmaN(-4) and RmaC as a basis for the design of caged intein.

### Prevention of unconditional protein splicing by caging Rma intein

Next, we designed the caged Rma DnaB intein based on the RmaN(-4) and RmaC, under the guidance of the amino acid sequence of other known caged inteins such as caged Npu DnaE intein^16,20^. A previous study using Npu DnaE intein reported that the protein splicing of split intein begins with the electrostatic interaction between the C-terminal anionic region of N-intein and the N-terminal cationic region of C-intein. This interaction triggers the formation of an intermediate structure, followed by the hydrophobic interaction between the N-terminal region of N-intein and the C-terminal region of C-intein to fold into a specific structure that is necessary to complete protein splicing^21^. Based on this folding mechanism, we designed the cage sequences for RmaN(-4) and RmaC using Clustal Omega for amino acid sequence alignment^22^ (Fig. S1). The caged RmaN(-4) (RmaN(-4)^cage^) was developed by adding amino acid residues 1–46 of RmaC to the C-terminal side of RmaN(-4). In addition, to investigate whether unconditional protein splicing of Rma DnaB intein can be inhibited by a shorter cage, we also added the amino acid residues 1–30 of RmaC to the C-terminal side of RmaN(-4) for developing the RmaN(-4) with short cage (RmaN(-4)^Scage^). The amino acid residues 1–30 of RmaC correspond to residues 1–13 of Npu C-intein (Fig. S1). This region of Npu C-intein was used as the first version of a cage for Npu N-intein, although it was insufficient to prevent unconditional protein splicing in the case of Npu DnaE intein^16^. Similarly, amino acid residues 51–102 of RmaN(-4) were added to the N-terminal side of RmaC to form the caged RmaC (RmaC^cage^) (Fig. 2d).

Then, pDNAs expressing the caged intein-fused C46-split CaVT was co-transfected into HeLa cells with pSV40-2xScMS2(C)-Luc2 and pNL1.1TK[Nluc/TK] to determine whether the designed cage could inhibit unconditional protein splicing of Rma DnaB intein. As expected, when the RmaN(-4)^cage^ (or Rma(-4)^Scage^) and the RmaC^cage^ were fused to the C46-split CaVT fragments as the vector for cell transfection, the Luc2 translation was not repressed, which means the caging successfully inhibits unconditional protein splicing of Rma DnaB intein (Figs. 2e,f, S2).

### Caged Rma DnaB intein fused with nanobody for conditional translational repression

**Figure 3:**
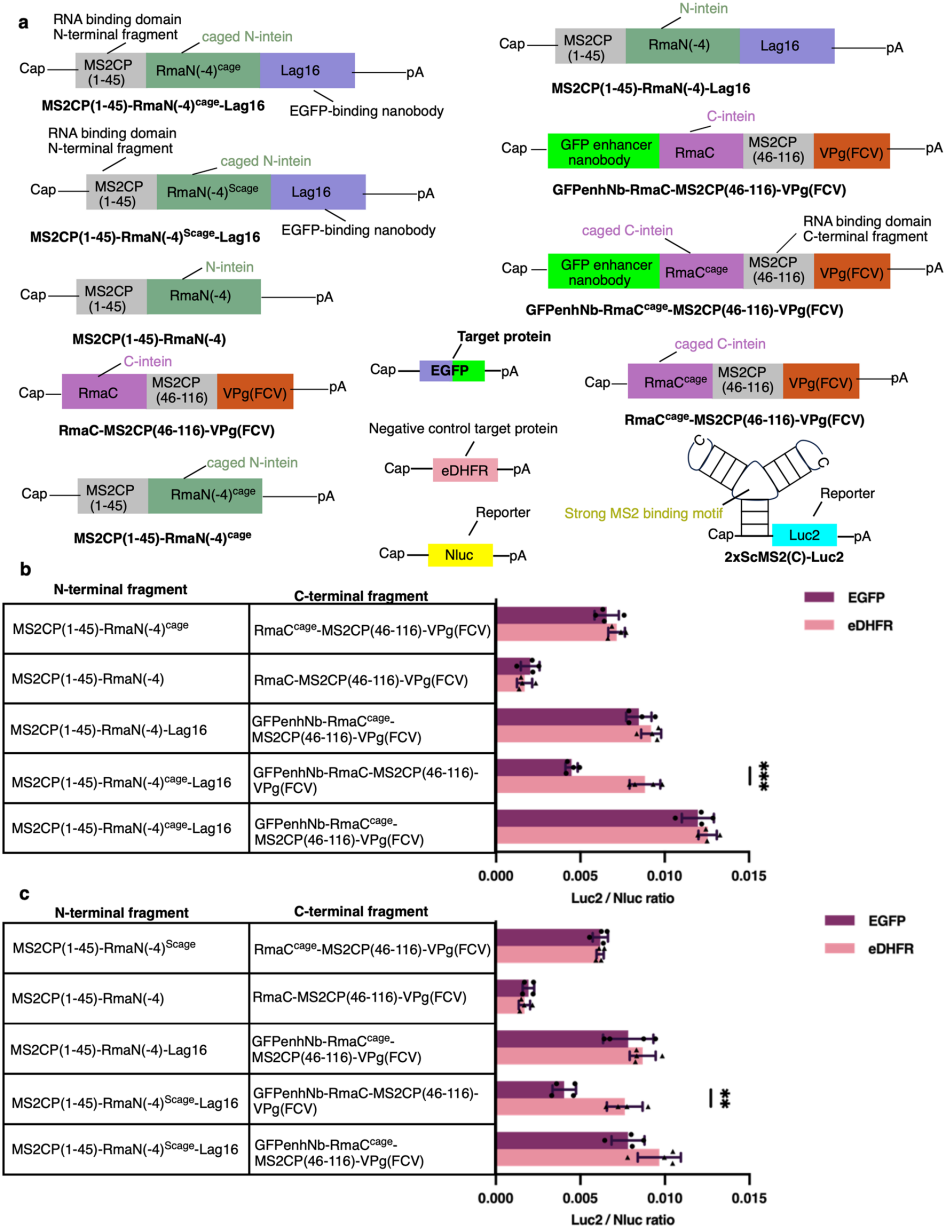
Conditional translational repression by the caged Rma DnaB intein-mediated reconstitution of EGFP-responsive C46-split CaVT. (**a**) Schematic depiction of mRNAs used in EGFP-responsive translational repression. (**b-c**) HeLa cells were co-transfected with 2xScMS2(C)-Luc2 (10 ng/well), Nluc (1 ng/well), and two types of mRNAs to express split CaVT per group (40 ng/well each), and EGFP or eDHFR (10 ng/well) mRNAs. The positive control for translational repression was MS2CP(1–45)-RmaN(-4) with RmaC-MS2CP(46–116)-VPg(FCV) group. The negative control for translational repression was MS2CP(1–45)-RmaN(-4)^cage^ or MS2CP(1–45)-RmaN(-4)^Scage^ with RmaC^cage^-MS2CP(46–116)-VPg(FCV) group. EGFP-responsive translational repression of Luc2 mRNA by RmaN(-4)^cage^ combined with the cage-free RmaC (**b**). EGFP-responsive translational repression of Luc2 mRNA by RmaN(-4)^Scage^ combined with the cage-free RmaC (**c**). The bar graph shows the Luc2/Nluc ratio (mean ± SD, n = 4). **P < 0.01, ***P < 0.001 compared with eDHFR-transfected (EGFP-untransfected) cells in each group by the unpaired two-sided Student’s t- test.

Next, to achieve target protein-responsive translational regulation, we constructed mRNAs to express fusion proteins of C46-split CaVT, the caged Rma DnaB intein, and nanobodies (Fig. S2). In the protein-responsive translational regulation system, two nanobodies must bind different epitopes of the same protein. Therefore, we selected two GFP-targeting nanobodies, GFP-enhancer nanobody^23^ and Lag16^24^, which were previously shown to bind different epitopes of GFP^4,25^. We additionally produced an artificial Luc2 mRNA harboring a strong MS2-binding motif at the 5′ UTR^4^ (Fig. 3a). The translational repression efficiency of these split CaVTs was analyzed based on the expression of Luc2 in mRNAs containing a strong MS2-binding motif.

When RmaC^cage^ was used as a component of EGFP-responsive C46-split CaVT, no conditional translational repression was observed. This may be due to the strong protein splicing inhibition by the designed cage, which prevents conditional protein splicing even in the presence of the target protein. In contrast, when RmaN(-4)^cage^ was combined with the cage-free RmaC, EGFP-responsive translational repression of Luc2 mRNA was induced (Fig. 3b). We also tested that EGFP-responsive translational repression could also be induced when RmaN(-4)^Scage^ was used instead of RmaN(-4)^cage^ (Fig. 3c). Since RmaN(-4)^cage^ shows stronger protein splicing inhibition than RmaN(-4)^Scage^ in the absence of target protein, we selected RmaN(-4)^cage^ for subsequent study.

### Construct a target protein-responsive translational regulation system containing two different sets of caged intein

**Figure 4:**
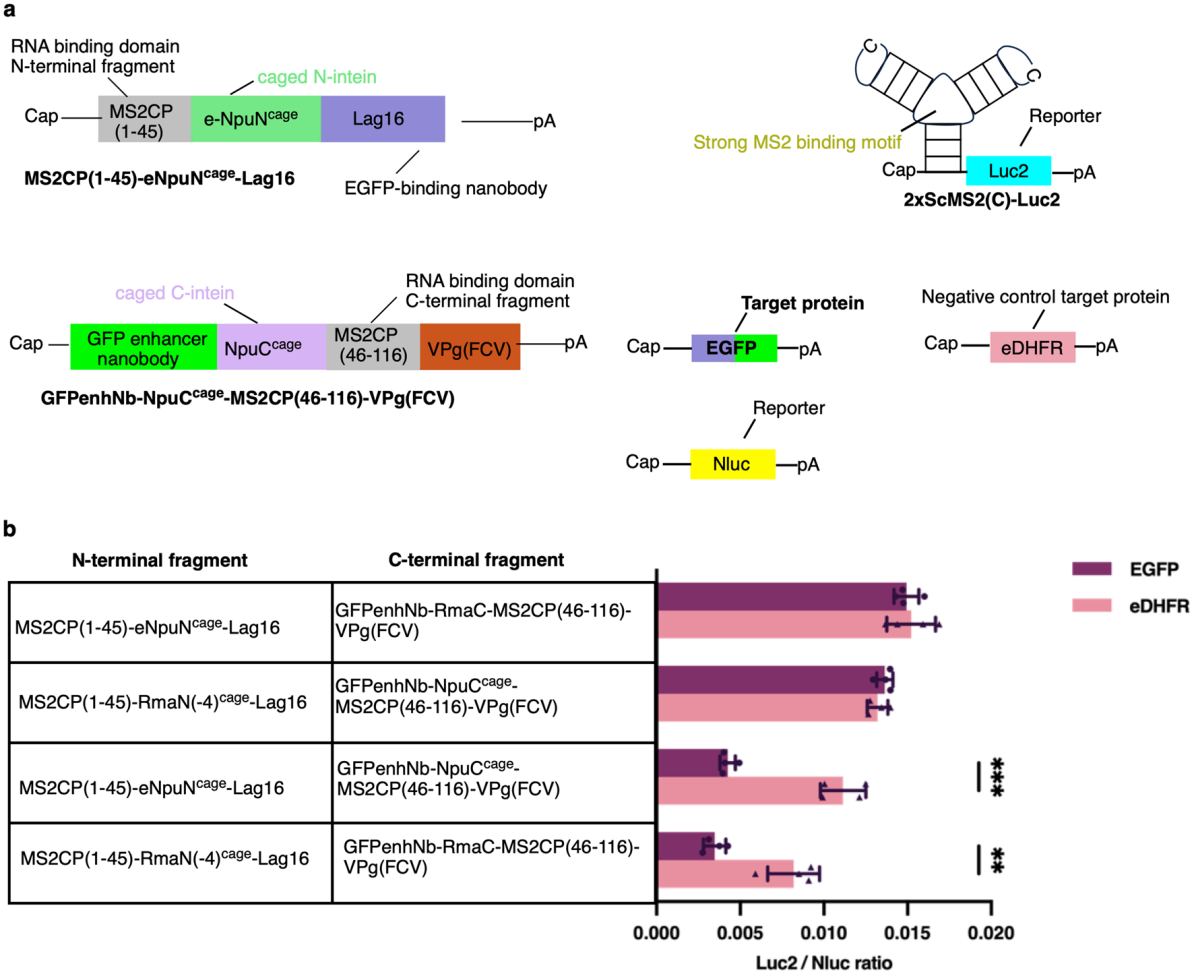
Orthogonality check of the caged Rma DnaB and Npu DnaE inteins by translational repression using EGFP-responsive C46-split CaVT. (**a**) Schematic depiction of mRNAs used in EGFP-responsive translational repression via caged Npu DnaE intein. (**b**) HeLa cells were co-transfected with 2xScMS2(C)-Luc2 (10 ng/well), Nluc (1 ng/well), and two types of mRNAs to express split CaVT per group, (40 ng/well each), and EGFP or eDHFR (10 ng/well) mRNAs. MS2CP(1–45)-eNpuN^cage^-Lag16 with GFPenhNb-NpuC^cage^-MS2CP(46–116)-VPg(FCV) group and MS2CP(1–45)-RmaN(-4)^cage^-Lag16 with GFPenhNb-RmaC-MS2CP(46–116)-VPg(FCV) group were used as positive controls for EGFP-responsive translational repression. The bar graph shows the Luc2/Nluc ratio (mean ± SD, n = 4). **P < 0.01, ***P < 0.001 compared with eDHFR-transfected (EGFP-untransfected) cells in each group by the unpaired two-sided Student’s t-test.

To achieve the multiple protein-responsive translational regulation, we planned to jointly use the caged Rma DnaB intein and the caged Npu DnaE intein in the same system. Prior to using these split caged intein pairs in the same application, we checked their orthogonality by transfecting EGFP-responsive C46-split CaVT containing either of these inteins (Figs. 3a, 4a). As shown in Fig. 4b, the combination of the caged Npu N-intein (eNpuN^cage^) and RmaC did not show EGFP-responsive protein splicing. Similar result was obtained by the combination of RmaN(-4)^cage^ and the caged Npu C-intein (NpuC^cage^). In contrast, when the caged N-inteins were combined with their original counterparts, EGFP-responsive protein splicing was observed. These results suggest that the two pairs of split caged inteins, the caged Rma DnaB and Npu DnaE inteins, are orthogonal and can be used in the same system without cross-reaction.

### Construct a logic gate using orthogonal caged split inteins for multi-input conditional translation regulation

**Figure 5:**
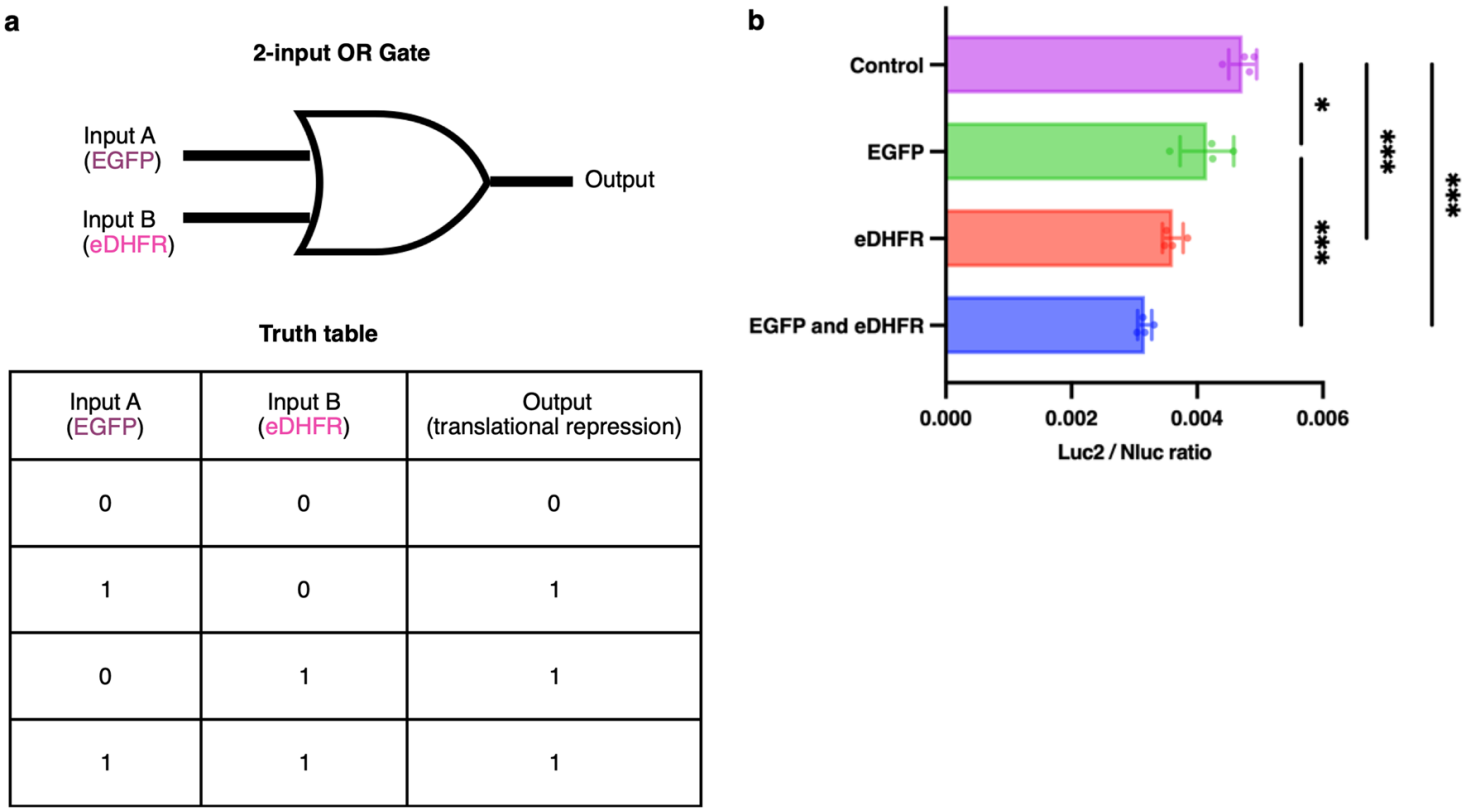
Translational repression by the reconstituted EGFP- and eDHFR-responsive C46-split CaVT via orthogonal caged split inteins that enable OR gate. (**a**) Schematic and truth table of mRNAs used in target protein-responsive translational repression via orthogonal caged split intein. (**b**) Cell transfection using orthogonal caged split inteins that enable OR gate. HeLa cells were co-transfected with 2xScMS2(C)-Luc2 (10 ng/well), Nluc (1 ng/well), MS2CP(1–45)-eNpuN^cage^-Nb113 (17.5 ng/well), CA1698-NpuC^cage^-MS2CP(46–116)-VPg(FCV) (17.5 ng/well), MS2CP(1–45)-RmaN(-4)^cage^-Lag16 (17.5 ng/well), GFPenhNb-RmaC-MS2CP(46–116)-VPg(FCV) (17.5 ng/well), and EGFP or eDHFR (10 ng/well) mRNAs, also human codon-optimized Barstar mRNA (10 ng/well) as negative target mRNA. The bar graph shows the Luc2/Nluc ratio (mean ± SD, n = 4). *P < 0.05, **P < 0.01, ***P < 0.001 compared with the negative control by Tukey’s multiple comparison test.

The utilization of split inteins has demonstrated their effectiveness in implementing cellular logic operations^19,26–30^. Therefore, we used two different sets of caged split inteins with C46-split CaVT to construct an OR gate (Fig. 5a) that enables simultaneous regulation of two different C46-split CaVT pairs to achieve translational regulation using two different intracellular proteins as inputs. In the OR gate, we used two sensor modules. One is the EGFP-responsive C46-split CaVT using the caged Rma DnaB intein, and the other is the *Escherichia coli* dihydrofolate reductase (eDHFR)-responsive C46-split CaVT using the caged Npu DnaE intein. The eDHFR-responsive C46-split CaVT uses eDHFR as a target protein. The N- and C-terminal fragments of it contain the eDHFR *α* epitope-targeting nanobody Nb113 and the *β* epitope-targeting nanobody CA1698^31^ respectively, and induced the reconstitution of full-length CaVT in the presence of eDHFR^4^. Thus, like EGFP-responsive C46-split CaVT, eDHFR-responsive C46-split CaVT induces translational repression in an eDHFR-dependent manner. When at least one of EGFP and eDHFR was present, Luc2 translation was repressed (Fig. 5b). The result indicates that the reconstitution of CaVT can be induced by both target proteins and demonstrates the successful creation of the OR gate.

## Discussion

In this study, we developed the caged Rma DnaB intein that enables conditional protein splicing. The attached cage successfully inhibited unconditional protein splicing of Rma DnaB intein and enabled target protein-dependent reconstitution of the full-length translational regulator protein from split fragments. The reconstituted translational regulator protein binds to target mRNA and induces translational repression in a target protein-dependent manner.

To achieve a target protein-responsive translational regulation system, we constructed mRNA to produce a fusion of C46-split CaVT, caged Rma DnaB intein, and nanobodies. Although caging both RmaN(-4) and RmaC efficiently inhibited unconditional protein splicing, the system was unable to induce EGFP-responsive translational repression even when the EGFP-targeting nanobodies were fused (Fig. 3). We considered that caging both RmaN(-4) and RmaC resulted in too strong inhibition of protein splicing to allow EGFP-responsive reconstitution of the full-length CaVT. Thus, we added cage to either RmaN(-4) or RmaC only to reduce the inhibitory effect on protein splicing. As anticipated, the combination of the caged RmaN(-4) and the cage-free RmaC resulted in EGFP-responsive translational repression, suggesting that the reconstitution of the full-length CaVT from split fragments by conditional protein splicing (Fig. 3). On the other hand, the combination of the cage-free RmaN(-4) and the caged RmaC failed to induce EGFP-responsive translational repression. One possible reason for the difference is the balance of the inteins and their cage portions. In RmaN(-4)^cage^, the length of the cage (68 amino acids) is 2/3 of RmaN(-4) (102 amino acids). On the other hand, in RmaC^cage^, the length of the cage (74 amino acids) is almost 3/2 of RmaC (51 amino acids). The relatively large cage may be a potent steric barrier for RmaC to interact with RmaN(-4), which does not allow protein splicing even in the presence of the target protein.

When constructing the multiple protein-responsive translational regulation system, we proved that the caged Rma DnaB intein and the caged Npu DnaE intein are orthogonal (Fig. 4). Thus, the caged Rma DnaB intein can be used in conjunction with the caged Npu DnaE intein to develop an mRNA-based logic gate for multi-input conditional translation. Compared to the caged Npu DnaE intein, the caged Rma DnaB intein showed lower fold change in C46-split CaVT-mediated translational regulation. This relatively lower protein splicing efficiency of the caged Rma DnaB intein may cause weaker translational repression in the EGFP-only group than in the eDHFR-only group in the OR gate that responds to both EGFP and eDHFR (Fig. 5). However, it should be noted that C46 was selected as a split site suitable for the caged Npu DnaE intein^4^. Different from Npu DnaE intein, the native residue downstream of Rma DnaB intein is not cysteine but serine^15^. Therefore, it is possible that there are better split sites than C46 for the caged Rma DnaB intein. Another strategy to improve the fold change is creating a time lag between the translational initiation of the regulatory component-encoding mRNAs and that of the target mRNA. Since these two types of mRNAs were simultaneously delivered in this study, the translation of the target mRNA should not be repressed until the regulatory components were sufficiently expressed even in the target protein-expressing cells. Thus, reducing such leaky translation by delaying the translational initiation of the target mRNA will improve the translational regulation efficiency. Although it is difficult to delay the translational initiation only by sequence engineering, there are several possible approaches such as the delayed delivery by a controlled release system or the addition of chemical cages that are removed by cytoplasmic enzymes to the target mRNA. The combination of such technologies and our translational regulation system may achieve further safer mRNA medicine in the future.

Utilizing the orthogonality of two caged inteins, we succeeded in constructing an mRNA-based logic gate, enabling multi-input conditional translation (Fig. 5). Such multi-input regulation is useful for selective translation of therapeutic proteins to develop safe and functional mRNA drugs since single-input is sometimes insufficient to distinguish cell types^9^.

The multiple protein-responsive translational regulation system developed in this study can be further improved and utilized. Prospects and applications include improving the safety of mRNA therapy and other gene therapies. Theoretically, for any therapeutic proteins of known sequence, mRNAs can be rapidly produced by in vitro enzymatic reactions, thus avoiding the complexity of manufacturing^8,32^. This indicates that we can select any therapeutic proteins and utilize synthetic mRNA for translation and regulation of proteins, which reflects the scalability of the system. Moreover, depending on the target protein we are interested in, the corresponding nanobody can be newly obtained by immunizing camelids and this will allow the target protein-responsive mRNA translational regulation system to be applied in a variety of target environments, thereby providing greater prospects for the development.

While we developed the CaVT-based translational regulation system in this study, other regulator proteins can also be utilized for protein-responsive translational regulation. Furthermore, caged inteins can be applied to various types of proteins other than translational regulators. For example, we have recently reported selective cell elimination by target protein-responsive reconstitution of the cytotoxic protein^33^. Selectable marker proteins^34^ and genome editing enzymes^27^ are other candidates for conditional reconstitution. The former may enable the selection of specific cells. The latter can be utilized for cell or condition-selective genome editing and its therapeutic applications. Finally, although our main purpose of developing the caged intein is its applications for mRNA therapy, it can be also a useful tool for other types of gene therapy (e.g., based on viral vectors or plasmid DNAs). Thus, the new caged split intein will open up more possibilities for future gene therapy.

## Methods

### Cell culture

HeLa cells were cultured in Dulbecco's Modified Eagle's Medium–high glucose (Sigma- Aldrich Japan, Tokyo, Japan) or D-MEM (High Glucose) with L-Glutamine and Phenol Red (Wako Pure Chemical Industries Ltd., Osaka, Japan) containing 10% fetal bovine serum and 1% penicillin–streptomycin (Sigma-Aldrich Japan) at 37 °C and 5% CO_2_.

### pDNA construction

KOD One PCR Master Mix (Toyobo Co., Ltd., Osaka, Japan) was used for the polymerase chain reaction (PCR) to prepare inserts. Oligo DNAs were purchased from Eurofins Genomics K.K. (Tokyo, Japan). Inserts and vectors digested with restriction enzymes were purified with the Monarch PCR & DNA Cleanup Kit. (New England BioLabs Japan Inc., Tokyo, Japan). The cloning reaction was performed using the In-Fusion HD Cloning Kit (Takara Bio, Shiga, Japan) or In-Fusion Snap Assembly Master Mix (Takara Bio). KAPA2G Fast HotStart ReadyMix with dye (2X) (Nippon Genetics Co., Ltd., Tokyo, Japan) or EmeraldAmp MAX PCR Master Mix (2 × Premix) (Takara Bio) was used for colony PCR. Plasmid DNAs were amplified in *E. coli* strain HST08 and purified using the QIAprep Spin Miniprep Kit (QIAGEN K.K., Japan). The concentration of purified plasmid DNAs was measured by NanoDrop One (Thermo Fisher Scientific K.K., Kanagawa, Japan). The sequences of constructed plasmid DNAs were analyzed by the Sanger sequencing service (Genewiz Japan Corp., Tokyo, Japan).

### pDNA transfection for luciferase assay

HeLa cells (1 × 10^4^) were seeded to a Corning 96-well Flat Clear Bottom White Polystyrene TC-treated Microplates (Corning Japan K.K., Tokyo, Japan). After 24 h of cell seeding, cells were transfected with 0.4 μL/well Lipofectamine LTX with Plus Reagent (Thermo Fisher Scientific K.K.) according to manufacturer’s instruction.

### In vitro transcription of mRNAs

Template DNAs for in vitro transcription were prepared by PCR using the PrimeSTAR Max DNA Polymerase (Takara Bio) and purified with the Monarch PCR & DNA Cleanup Kit. Then MEGAscript T7 Transcription Kit (Thermo Fisher Scientific K.K.) which contains GTP, CTP, and ATP was used for in vitro transcription reaction. *N*^1^-methyl-pseudo-UTP (TriLink Biotechnologies, San Diego, CA, USA) and CleanCap Reagent AG (TriLink Biotechnologies) were also used for reaction of in vitro transcription. Transcripts were treated with Turbo DNase (Thermo Fisher Scientific K.K.) and purified by RNeasy Mini Kit (Qiagen K.K., Tokyo, Japan). Then, the obtained mRNAs were dephosphorylated using Quick CIP (New England BioLabs Japan) and purified using the RNeasy Mini Kit. The concentration of purified mRNAs was quantified by NanoDrop One. The mRNAs were analyzed using the Agilent RNA 6000 Nano Assay and the Agilent 2100 Bioanalyzer (Agilent Technologies Japan Ltd., Tokyo, Japan).

### mRNA transfection

HeLa cells (1 × 10^4^) were seeded to a Corning 96-well Flat Clear Bottom White Polystyrene TC-treated Microplates (Corning Japan K.K.). After 24 h of cell seeding, cells were transfected with 0.3 μL/well Lipofectamine MessengerMAX (Thermo Fisher Scientific K.K.) according to manufacturer’s instruction.

### Dual luciferase assay

The luminescence of Luc2 and Nluc was measured 24 h after transfection using a Nano-Glo Dual-Luciferase Assay System (Promega K.K., Tokyo, Japan) and a GloMax Navigator microplate luminometer (Promega K.K.).

### Supplementary Information

Supplementary Information.

## Data Availability

The datasets generated during and/or analysed during the current study are available from the corresponding author on reasonable request.
