# Supplementary material for: Development of a new caged intein for multi-input conditional translation of synthetic mRNA
Source: Sci Rep. 2024 May 1;14:9988. doi: 10.1038/s41598-024-60809-w (PMC11063168; doi:10.1038/s41598-024-60809-w)
Supplement: Supplementary file 1 — Supplementary Information. [file 41598_2024_60809_MOESM1_ESM.docx]

**Development of a new caged intein for multi-input conditional translation of synthetic mRNA**

**Tingting Yang^1^, Hideyuki Nakanishi^1,2^* and Keiji Itaka^1,2^***

^1^Department of Biofunction Research, Institute of Biomaterials and Bioengineering, Tokyo Medical and Dental University (TMDU), Tokyo, 101-0062, Japan

^2^Center for Infectious Disease Education and Research (CiDER), Osaka University, Osaka, 565-0871, Japan

*Corresponding: nakanishi.hideyuki.3m@kyoto-u.jp(H.N.);itaka.bif@tmd.ac.jp(K.I.).

Supplementary Figure

- Figure S1. Sequence alignment of Rma DnaB intein with Npu DnaE intein.
- Figure S2. Schematic diagram illustrating outcomes of each C46-split CaVT pair.

Supplementary Materials and Methods

- Coding region sequences of split CaVT expression vectors.
- List of primers used to prepare template DNAs for in vitro transcription.
- Full sequences of template DNAs for in vitro transcription.


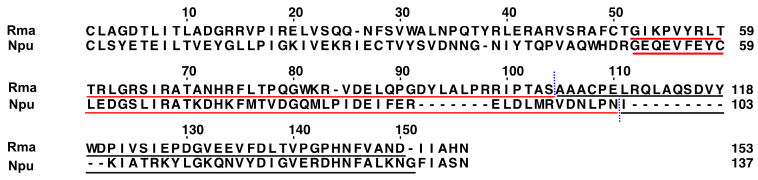


**Figure S1.** Sequence alignment of Rma DnaB intein with Npu DnaE intein. The amino acid sequences of RmaN(-4)-RmaC fusion and NpuN-NpuC fusion are aligned. Underlined in red are the regions of N-inteins utilized to make the cages for C-inteins, while underlined in black are the regions of C-inteins utilized to make the cages for N-intein. The blue dashed line indicates the boundary of N- and C-inteins.


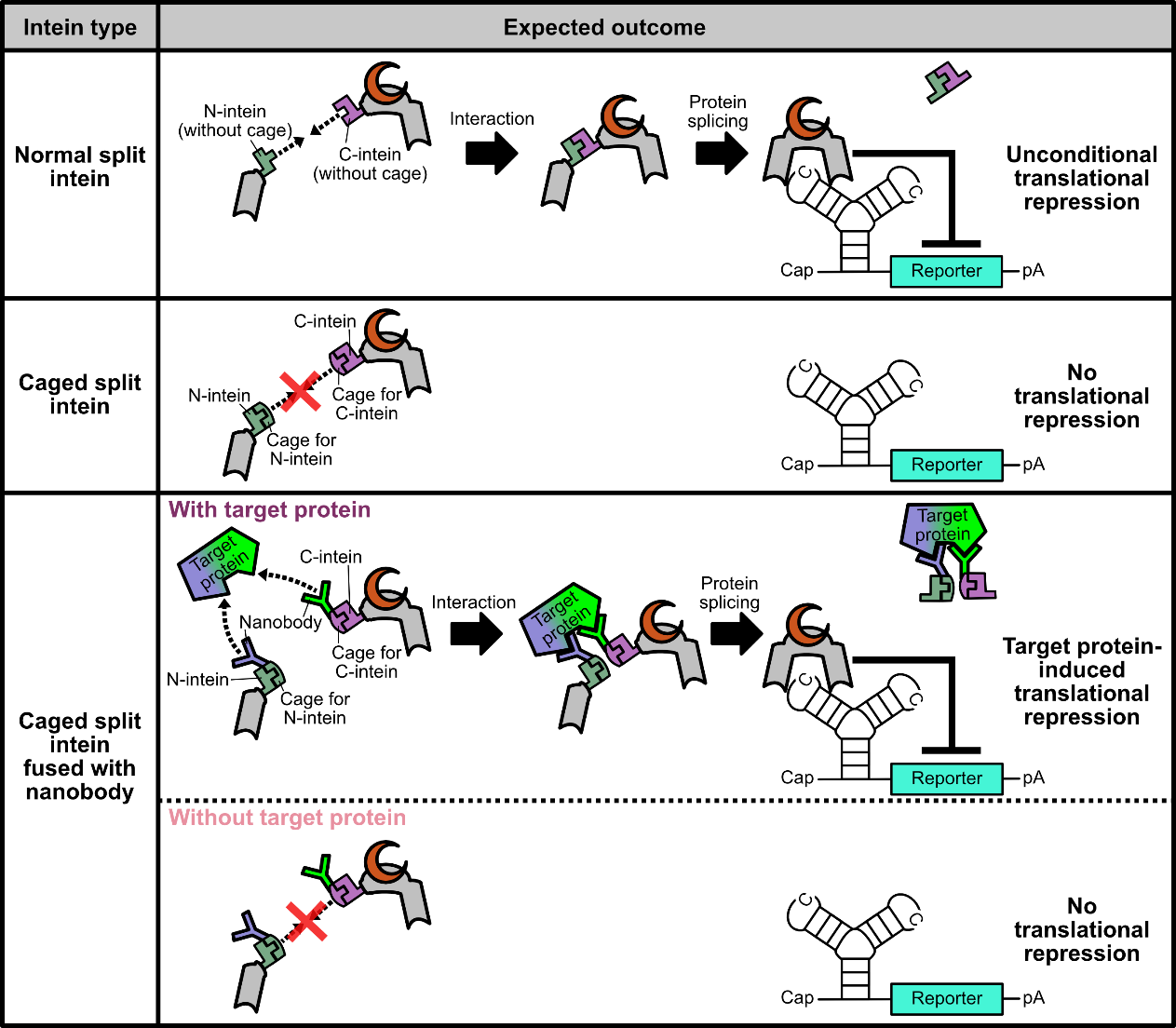
**Figure S2.** Schematic diagram illustrating outcomes of each C46-split CaVT pair.

**Coding region sequences of split CaVT expression vectors**

- **pcDNA3.1-MS2CP(1-45)-RmaN**

Start codon: 1-3, MS2CP N-terminal fragment gene: 4-138, Rma N-intein gene: 139- 456, DYK-tag gene: 475-498, Stop codon: 499-501

[ATGGCTTCTAACTTTACTCAGTTCGTTCTCGTCGACAATGGCGGAACTGGCGACGTGACTGTCGCCCCAAGCAACTTCGCTAACGGGGTCGCTGAATGGATCAGCTCTAACTCGCGATCACAGGCTTACAAAGTAACCTGTCTGGCCGGCGATACCCTGATCACCCTGGCCGATGGCAGAAGAGTGCCCATCCGCGAGCTGGTGTCCCAGCAGAACTTTAGCGTGTGGGCCCTGAACCCCCAGACCTACAGACTGGAACGGGCCAGAGTGTCCCGGGCCTTCTGTACCGGAATCAAGCCCGTGTACCGGCTGACCACACGGCTGGGCAGATCCATCAGAGCCACCGCCAACCACCGGTTTCTGACACCCCAGGGCTGGAAGAGAGTGGACGAACTGCAGCCCGGCGACTACCTGGCCCTGCCCAGAAGAATCCCTACCGCCAGCACCCCCACCCTGACCGGTGTTCCAACTGTTGATTACAAGGATGACGACGATAAGTGA]

- **pcDNA3.1-MS2CP(1-45)-RmaN(-4)**

Start codon: 1-3, MS2CP N-terminal fragment gene: 4-138, Rma N-intein gene: 139- 444, DYK-tag gene: 463-486, Stop codon: 487-489

[ATGGCTTCTAACTTTACTCAGTTCGTTCTCGTCGACAATGGCGGAACTGGCGACGTGACTGTCGCCCCAAGCAACTTCGCTAACGGGGTCGCTGAATGGATCAGCTCTAACTCGCGATCACAGGCTTACAAAGTAACCTGTCTGGCCGGCGATACCCTGATCACCCTGGCCGATGGCAGAAGAGTGCCCATCCGCGAGCTGGTGTCCCAGCAGAACTTTAGCGTGTGGGCCCTGAACCCCCAGACCTACAGACTGGAACGGGCCAGAGTGTCCCGGGCCTTCTGTACCGGAATCAAGCCCGTGTACCGGCTGACCACACGGCTGGGCAGATCCATCAGAGCCACCGCCAACCACCGGTTTCTGACACCCCAGGGCTGGAAGAGAGTGGACGAACTGCAGCCCGGCGACTACCTGGCCCTGCCCAGAAGAATCCCTACCGCCAGCACCGGTGTTCCAACTGTTGATTACAAGGATGACGACGATAAGTGA]

- **pcDNA3.1-RmaC-MS2CP(46-116)-VPg(FCV)**

Start codon: 1-3, Rma C-intein gene: 4-156, MS2CP C-terminal fragment gene: 157-369, FCV-derived VPg gene: 376-708, DYK-tag gene: 727-750, Stop codon: 751-753

[ATGGCCGCTGCCTGCCCTGAGCTGAGACAGCTGGCACAGAGCGACGTGTACTGGGACCCCATCGTGTCCATCGAGCCCGACGGCGTGGAAGAGGTGTTCGACCTGACAGTGCCTGGCCCCCACAACTTCGTGGCCAACGACATCATTGCCCACAACTGTAGCGTTCGTCAGAGCTCTGCGCAGAATCGCAAATACACCATCAAAGTCGAGGTGCCTAAAGGCGCATGGAGGTCTTACTTAAATATGGAACTAACCATTCCAATTTTCGCCACGAATTCCGACTGCGAGCTTATTGTTAAGGCAATGCAAGGTCTCCTAAAAGATGGAAACCCGATTCCCTCGGCCATCGCGGCCAACTCCGGCATCTACGGATCCGCCAAGGGCAAGACCAAGAGCAAAGTGGGCCCCTACAGAGGCAGAGGCGTGGCCCTTACAGACGACGAGTATGACGAATGGCGCGAGCACAACGCCACCAGAAAGCTGGATCTGAGCGTGGAAGATTTCCTGATGCTGCGGCACAGAGCCGCTCTGGGAGCTGATGATGCCGACGCCGTGAAGTTCAGATCCTGGTGGAACAGCAGAAGCCGGCTGGCCGACGATTACGAGGATGTGACCGTGATCGGCAAAGGCGGCGTGAAGCACGAGAAGATCCGGACCAATACTCTGAGAGCCGTGGACAGAGGCTACGACGTGTCCTTCGCTGAAGAAACCGGTGTTCCAACTGTTGATTACAAGGATGACGACGATAAGTGA]

- **pcDNA3.1-MS2CP(1-45)-RmaN(-4)^cage^**

Start codon: 1-3, MS2CP N-terminal fragment gene: 4-138, Caged Rma N-intein gene: 139- 648, DYK-tag gene: 667-690, Stop codon: 691-693

[ATGGCTTCTAACTTTACTCAGTTCGTTCTCGTCGACAATGGCGGAACTGGCGACGTGACTGTCGCCCCAAGCAACTTCGCTAACGGGGTCGCTGAATGGATCAGCTCTAACTCGCGATCACAGGCTTACAAAGTAACCTGTCTGGCCGGCGATACCCTGATCACCCTGGCCGATGGCAGAAGAGTGCCCATCCGCGAGCTGGTGTCCCAGCAGAACTTTAGCGTGTGGGCCCTGAACCCCCAGACCTACAGACTGGAACGGGCCAGAGTGTCCCGGGCCTTCTGTACCGGAATCAAGCCCGTGTACCGGCTGACCACACGGCTGGGCAGATCCATCAGAGCCACCGCCAACCACCGGTTTCTGACACCCCAGGGCTGGAAGAGAGTGGACGAACTGCAGCCCGGCGACTACCTGGCCCTGCCCAGAAGAATCCCTACCGCCAGCGGCAGCGGCGGCGAGAACCTGTACTTCCAGGGCGAGAACCTGTACTTCCAGGGCGGCAGCGGCGGCGCCGCTGCCTGCCCTGAGCTGAGACAGCTGGCACAGAGCGACGTGTACTGGGACCCCATCGTGTCCATCGAGCCCGACGGCGTGGAAGAGGTGTTCGACCTGACAGTGCCTGGCCCCCACAACTTCGTGGCCAACGACACCGGTGTTCCAACTGTTGATTACAAGGATGACGACGATAAGTGA]

- **pcDNA3.1-MS2CP(1-45)-RmaN(-4)^Scage^**

Start codon: 1-3, MS2CP N-terminal fragment gene: 4-138, Caged Rma N-intein gene: 139- 600, DYK-tag gene: 619-642, Stop codon: 643-645

[ATGGCTTCTAACTTTACTCAGTTCGTTCTCGTCGACAATGGCGGAACTGGCGACGTGACTGTCGCCCCAAGCAACTTCGCTAACGGGGTCGCTGAATGGATCAGCTCTAACTCGCGATCACAGGCTTACAAAGTAACCTGTCTGGCCGGCGATACCCTGATCACCCTGGCCGATGGCAGAAGAGTGCCCATCCGCGAGCTGGTGTCCCAGCAGAACTTTAGCGTGTGGGCCCTGAACCCCCAGACCTACAGACTGGAACGGGCCAGAGTGTCCCGGGCCTTCTGTACCGGAATCAAGCCCGTGTACCGGCTGACCACACGGCTGGGCAGATCCATCAGAGCCACCGCCAACCACCGGTTTCTGACACCCCAGGGCTGGAAGAGAGTGGACGAACTGCAGCCCGGCGACTACCTGGCCCTGCCCAGAAGAATCCCTACCGCCAGCGGCAGCGGCGGCGAGAACCTGTACTTCCAGGGCGAGAACCTGTACTTCCAGGGCGGCAGCGGCGGCGCCGCTGCCTGCCCTGAGCTGAGACAGCTGGCACAGAGCGACGTGTACTGGGACCCCATCGTGTCCATCGAGCCCGACGGCGTGGAAGAGACCGGTGTTCCAACTGTTGATTACAAGGATGACGACGATAAGTGA]

- **pcDNA3.1-RmaC^cage^-MS2CP(46-116)-VPg(FCV)**

Start codon: 1-3, Caged Rma C-intein gene: 4-378, MS2CP C-terminal fragment gene: 379-591, FCV-derived VPg gene: 598-930, DYK-tag gene: 949-972, Stop codon: 973-975

[ATGGGAATCAAGCCCGTGTACCGGCTGACCACACGGCTGGGCAGATCCATCAGAGCCACCGCCAACCACCGGTTTCTGACACCCCAGGGCTGGAAGAGAGTGGACGAACTGCAGCCCGGCGACTACCTGGCCCTGCCCAGAAGAATCCCTACCGCCAGCGGCAGCGGCGGCGAGAACCTGTACTTCCAGGGCGAGAACCTGTACTTCCAGGGCGGCAGCGGCGGCGCCGCTGCCTGCCCTGAGCTGAGACAGCTGGCACAGAGCGACGTGTACTGGGACCCCATCGTGTCCATCGAGCCCGACGGCGTGGAAGAGGTGTTCGACCTGACAGTGCCTGGCCCCCACAACTTCGTGGCCAACGACATCATTGCCCACAACTGTAGCGTTCGTCAGAGCTCTGCGCAGAATCGCAAATACACCATCAAAGTCGAGGTGCCTAAAGGCGCATGGAGGTCTTACTTAAATATGGAACTAACCATTCCAATTTTCGCCACGAATTCCGACTGCGAGCTTATTGTTAAGGCAATGCAAGGTCTCCTAAAAGATGGAAACCCGATTCCCTCGGCCATCGCGGCCAACTCCGGCATCTACGGATCCGCCAAGGGCAAGACCAAGAGCAAAGTGGGCCCCTACAGAGGCAGAGGCGTGGCCCTTACAGACGACGAGTATGACGAATGGCGCGAGCACAACGCCACCAGAAAGCTGGATCTGAGCGTGGAAGATTTCCTGATGCTGCGGCACAGAGCCGCTCTGGGAGCTGATGATGCCGACGCCGTGAAGTTCAGATCCTGGTGGAACAGCAGAAGCCGGCTGGCCGACGATTACGAGGATGTGACCGTGATCGGCAAAGGCGGCGTGAAGCACGAGAAGATCCGGACCAATACTCTGAGAGCCGTGGACAGAGGCTACGACGTGTCCTTCGCTGAAGAAACCGGTGTTCCAACTGTTGATTACAAGGATGACGACGATAAGTGA]

**List of primers used to prepare template DNAs for in vitro transcription.**

| Primer Name | Primer Sequence |
| --- | --- |
| HNT-34 | CAGTGAATTGTAATACGACTCACTATAAGGCGAATTAAGAGAGAAAAGAAGAG |
| HNT-9 | TTTTTTTTTTTTTTTTTTTTTTTTTTTTTTTTTTTTTTTTTTTTTTTTTTTTTTTTTTTTTTTTTTTTTTTTTTTTTTTTTTTTTTTTTTTTTTTTTTTTTTTTTTTTTTTTTTTTTTTTCCTACTCAGGCTTTATTCA |

**Full sequences of template DNAs for in vitro transcription**

- **MS2CP(1-45)-RmaN(-4)**

Template pDNA: pUTR2-MS2CP(1-45)-RmaN(-4)

Primers: HNT-34 and HNT-9

T7 promoter (for CleanCap AG Reagent): 11-30, Kozak sequence (including start codon): 79-87, MS2CP N-terminal fragment gene: 88-222, Rma N-intein gene: 223-528, Stop codon: 529-531

[CAGTGAATTGTAATACGACTCACTATAAGGCGAATTAAGAGAGAAAAGAAGAGTAAGAAGAAATATAAGACACCGGTCGCCACCATGGCTTCTAACTTTACTCAGTTCGTTCTCGTCGACAATGGCGGAACTGGCGACGTGACTGTCGCCCCAAGCAACTTCGCTAACGGGGTCGCTGAATGGATCAGCTCTAACTCGCGATCACAGGCTTACAAAGTAACCTGTCTGGCCGGCGATACCCTGATCACCCTGGCCGATGGCAGAAGAGTGCCCATCCGCGAGCTGGTGTCCCAGCAGAACTTTAGCGTGTGGGCCCTGAACCCCCAGACCTACAGACTGGAACGGGCCAGAGTGTCCCGGGCCTTCTGTACCGGAATCAAGCCCGTGTACCGGCTGACCACACGGCTGGGCAGATCCATCAGAGCCACCGCCAACCACCGGTTTCTGACACCCCAGGGCTGGAAGAGAGTGGACGAACTGCAGCCCGGCGACTACCTGGCCCTGCCCAGAAGAATCCCTACCGCCAGCTGAATCTAGACCTTCTGCGGGGCTTGCCTTCTGGCCATGCCCTTCTTCTCTCCCTTGCACCTGTACCTCTTGGTCTTTGAATAAAGCCTGAGTAGGAAAAAAAAAAAAAAAAAAAAAAAAAAAAAAAAAAAAAAAAAAAAAAAAAAAAAAAAAAAAAAAAAAAAAAAAAAAAAAAAAAAAAAAAAAAAAAAAAAAAAAAAAAAAAAAAAAAAAAAA]

- **MS2CP(1-45)-RmaN(-4)^cage^**

Template pDNA: pUTR2-MS2CP(1-45)-RmaN(-4)^cage^

Primers: HNT-34 and HNT-9

T7 promoter (for CleanCap AG Reagent): 11-30, Kozak sequence (including start codon): 79-87, MS2CP N-terminal fragment gene: 88-222, Caged Rma N-intein gene: 223-732, Stop codon: 733-735

[CAGTGAATTGTAATACGACTCACTATAAGGCGAATTAAGAGAGAAAAGAAGAGTAAGAAGAAATATAAGACACCGGTCGCCACCATGGCTTCTAACTTTACTCAGTTCGTTCTCGTCGACAATGGCGGAACTGGCGACGTGACTGTCGCCCCAAGCAACTTCGCTAACGGGGTCGCTGAATGGATCAGCTCTAACTCGCGATCACAGGCTTACAAAGTAACCTGTCTGGCCGGCGATACCCTGATCACCCTGGCCGATGGCAGAAGAGTGCCCATCCGCGAGCTGGTGTCCCAGCAGAACTTTAGCGTGTGGGCCCTGAACCCCCAGACCTACAGACTGGAACGGGCCAGAGTGTCCCGGGCCTTCTGTACCGGAATCAAGCCCGTGTACCGGCTGACCACACGGCTGGGCAGATCCATCAGAGCCACCGCCAACCACCGGTTTCTGACACCCCAGGGCTGGAAGAGAGTGGACGAACTGCAGCCCGGCGACTACCTGGCCCTGCCCAGAAGAATCCCTACCGCCAGCGGCAGCGGCGGCGAGAACCTGTACTTCCAGGGCGAGAACCTGTACTTCCAGGGCGGCAGCGGCGGCGCCGCTGCCTGCCCTGAGCTGAGACAGCTGGCACAGAGCGACGTGTACTGGGACCCCATCGTGTCCATCGAGCCCGACGGCGTGGAAGAGGTGTTCGACCTGACAGTGCCTGGCCCCCACAACTTCGTGGCCAACGACTGAATCTAGACCTTCTGCGGGGCTTGCCTTCTGGCCATGCCCTTCTTCTCTCCCTTGCACCTGTACCTCTTGGTCTTTGAATAAAGCCTGAGTAGGAAAAAAAAAAAAAAAAAAAAAAAAAAAAAAAAAAAAAAAAAAAAAAAAAAAAAAAAAAAAAAAAAAAAAAAAAAAAAAAAAAAAAAAAAAAAAAAAAAAAAAAAAAAAAAAAAAAAAAAA]

- **RmaC-MS2CP(46-116)-VPg(FCV)**

Template pDNA: pUTR2-RmaC-MS2CP(46-116)-VPg(FCV)

Primers: HNT-34 and HNT-9

T7 promoter (for CleanCap AG Reagent): 11-30, Kozak sequence (including start codon): 79-87, Rma C-intein gene: 88-240, MS2CP C-terminal fragment gene: 241-453, FCV-derived VPg gene: 460-792, Stop codon: 793-795

[CAGTGAATTGTAATACGACTCACTATAAGGCGAATTAAGAGAGAAAAGAAGAGTAAGAAGAAATATAAGACACCGGTCGCCACCATGGCCGCTGCCTGCCCTGAGCTGAGACAGCTGGCACAGAGCGACGTGTACTGGGACCCCATCGTGTCCATCGAGCCCGACGGCGTGGAAGAGGTGTTCGACCTGACAGTGCCTGGCCCCCACAACTTCGTGGCCAACGACATCATTGCCCACAACTGTAGCGTTCGTCAGAGCTCTGCGCAGAATCGCAAATACACCATCAAAGTCGAGGTGCCTAAAGGCGCATGGAGGTCTTACTTAAATATGGAACTAACCATTCCAATTTTCGCCACGAATTCCGACTGCGAGCTTATTGTTAAGGCAATGCAAGGTCTCCTAAAAGATGGAAACCCGATTCCCTCGGCCATCGCGGCCAACTCCGGCATCTACGGATCCGCCAAGGGCAAGACCAAGAGCAAAGTGGGCCCCTACAGAGGCAGAGGCGTGGCCCTTACAGACGACGAGTATGACGAATGGCGCGAGCACAACGCCACCAGAAAGCTGGATCTGAGCGTGGAAGATTTCCTGATGCTGCGGCACAGAGCCGCTCTGGGAGCTGATGATGCCGACGCCGTGAAGTTCAGATCCTGGTGGAACAGCAGAAGCCGGCTGGCCGACGATTACGAGGATGTGACCGTGATCGGCAAAGGCGGCGTGAAGCACGAGAAGATCCGGACCAATACTCTGAGAGCCGTGGACAGAGGCTACGACGTGTCCTTCGCTGAAGAATGAATCTAGACCTTCTGCGGGGCTTGCCTTCTGGCCATGCCCTTCTTCTCTCCCTTGCACCTGTACCTCTTGGTCTTTGAATAAAGCCTGAGTAGGAAAAAAAAAAAAAAAAAAAAAAAAAAAAAAAAAAAAAAAAAAAAAAAAAAAAAAAAAAAAAAAAAAAAAAAAAAAAAAAAAAAAAAAAAAAAAAAAAAAAAAAAAAAAAAAAAAAAAAAA]

- **RmaC^cage^-MS2CP(46-116)-VPg(FCV)**

Template pDNA: pUTR2-RmaC^cage^-MS2CP(46-116)-VPg(FCV)

Primers: HNT-34 and HNT-9

T7 promoter (for CleanCap AG Reagent): 11-30, Kozak sequence (including start codon): 79-87, Caged Rma C-intein gene: 88-462, MS2CP C-terminal fragment gene: 463-675, FCV-derived VPg gene: 682-1014, Stop codon: 1015-1017

[CAGTGAATTGTAATACGACTCACTATAAGGCGAATTAAGAGAGAAAAGAAGAGTAAGAAGAAATATAAGACACCGGTCGCCACCATGGGAATCAAGCCCGTGTACCGGCTGACCACACGGCTGGGCAGATCCATCAGAGCCACCGCCAACCACCGGTTTCTGACACCCCAGGGCTGGAAGAGAGTGGACGAACTGCAGCCCGGCGACTACCTGGCCCTGCCCAGAAGAATCCCTACCGCCAGCGGCAGCGGCGGCGAGAACCTGTACTTCCAGGGCGAGAACCTGTACTTCCAGGGCGGCAGCGGCGGCGCCGCTGCCTGCCCTGAGCTGAGACAGCTGGCACAGAGCGACGTGTACTGGGACCCCATCGTGTCCATCGAGCCCGACGGCGTGGAAGAGGTGTTCGACCTGACAGTGCCTGGCCCCCACAACTTCGTGGCCAACGACATCATTGCCCACAACTGTAGCGTTCGTCAGAGCTCTGCGCAGAATCGCAAATACACCATCAAAGTCGAGGTGCCTAAAGGCGCATGGAGGTCTTACTTAAATATGGAACTAACCATTCCAATTTTCGCCACGAATTCCGACTGCGAGCTTATTGTTAAGGCAATGCAAGGTCTCCTAAAAGATGGAAACCCGATTCCCTCGGCCATCGCGGCCAACTCCGGCATCTACGGATCCGCCAAGGGCAAGACCAAGAGCAAAGTGGGCCCCTACAGAGGCAGAGGCGTGGCCCTTACAGACGACGAGTATGACGAATGGCGCGAGCACAACGCCACCAGAAAGCTGGATCTGAGCGTGGAAGATTTCCTGATGCTGCGGCACAGAGCCGCTCTGGGAGCTGATGATGCCGACGCCGTGAAGTTCAGATCCTGGTGGAACAGCAGAAGCCGGCTGGCCGACGATTACGAGGATGTGACCGTGATCGGCAAAGGCGGCGTGAAGCACGAGAAGATCCGGACCAATACTCTGAGAGCCGTGGACAGAGGCTACGACGTGTCCTTCGCTGAAGAATGAATCTAGACCTTCTGCGGGGCTTGCCTTCTGGCCATGCCCTTCTTCTCTCCCTTGCACCTGTACCTCTTGGTCTTTGAATAAAGCCTGAGTAGGAAAAAAAAAAAAAAAAAAAAAAAAAAAAAAAAAAAAAAAAAAAAAAAAAAAAAAAAAAAAAAAAAAAAAAAAAAAAAAAAAAAAAAAAAAAAAAAAAAAAAAAAAAAAAAAAAAAAAAAA]

- **MS2CP(1-45)-RmaN(-4)-Lag16**

Template pDNA: pUTR2-MS2CP(1-45)-RmaN(-4)-Lag16

Primers: HNT-34 and HNT-9

T7 promoter (for CleanCap AG Reagent): 11-30, Kozak sequence (including start codon): 79-87, MS2CP N-terminal fragment gene: 88-222, Rma N-intein gene: 223-528, anti-GFP nanobody Lag16 gene: 535-915, Stop codon: 916-918

[CAGTGAATTGTAATACGACTCACTATAAGGCGAATTAAGAGAGAAAAGAAGAGTAAGAAGAAATATAAGACACCGGTCGCCACCATGGCTTCTAACTTTACTCAGTTCGTTCTCGTCGACAATGGCGGAACTGGCGACGTGACTGTCGCCCCAAGCAACTTCGCTAACGGGGTCGCTGAATGGATCAGCTCTAACTCGCGATCACAGGCTTACAAAGTAACCTGTCTGGCCGGCGATACCCTGATCACCCTGGCCGATGGCAGAAGAGTGCCCATCCGCGAGCTGGTGTCCCAGCAGAACTTTAGCGTGTGGGCCCTGAACCCCCAGACCTACAGACTGGAACGGGCCAGAGTGTCCCGGGCCTTCTGTACCGGAATCAAGCCCGTGTACCGGCTGACCACACGGCTGGGCAGATCCATCAGAGCCACCGCCAACCACCGGTTTCTGACACCCCAGGGCTGGAAGAGAGTGGACGAACTGCAGCCCGGCGACTACCTGGCCCTGCCCAGAAGAATCCCTACCGCCAGCGGATCCGCTCAGGTGCAGCTGGTGGAATCTGGTGGTAGACTGGTGCAGGCCGGCGATAGCCTGAGACTGTCTTGTGCCGCCAGCGGCAGAACCTTTAGCACATCTGCCATGGCCTGGTTCAGACAGGCCCCTGGCAGAGAAAGGGAATTCGTGGCCGCCATCACATGGACCGTGGGCAATACCATCCTGGGCGACTCTGTGAAGGGCAGATTCACCATCAGCCGGGACAGAGCCAAGAACACCGTGGACCTGCAGATGGACAACCTGGAACCTGAGGACACCGCCGTGTACTACTGCAGCGCCAGATCTAGAGGCTACGTGCTGTCCGTGCTGAGAAGCGTGGACAGCTACGATTATTGGGGCCAGGGCACCCAAGTGACCGTGTCTTGAATCTAGACCTTCTGCGGGGCTTGCCTTCTGGCCATGCCCTTCTTCTCTCCCTTGCACCTGTACCTCTTGGTCTTTGAATAAAGCCTGAGTAGGAAAAAAAAAAAAAAAAAAAAAAAAAAAAAAAAAAAAAAAAAAAAAAAAAAAAAAAAAAAAAAAAAAAAAAAAAAAAAAAAAAAAAAAAAAAAAAAAAAAAAAAAAAAAAAAAAAAAAAAA]

- **MS2CP(1-45)-RmaN(-4)^cage^-Lag16**

Template pDNA: pUTR2-MS2CP(1-45)-RmaN(-4)^cage^-Lag16

Primers: HNT-34 and HNT-9

T7 promoter (for CleanCap AG Reagent): 11-30, Kozak sequence (including start codon): 79-87, MS2CP N-terminal fragment gene: 88-222, Caged Rma N-intein gene: 223-732, anti-GFP nanobody Lag16 gene: 739-1119, Stop codon: 1120-1122

[CAGTGAATTGTAATACGACTCACTATAAGGCGAATTAAGAGAGAAAAGAAGAGTAAGAAGAAATATAAGACACCGGTCGCCACCATGGCTTCTAACTTTACTCAGTTCGTTCTCGTCGACAATGGCGGAACTGGCGACGTGACTGTCGCCCCAAGCAACTTCGCTAACGGGGTCGCTGAATGGATCAGCTCTAACTCGCGATCACAGGCTTACAAAGTAACCTGTCTGGCCGGCGATACCCTGATCACCCTGGCCGATGGCAGAAGAGTGCCCATCCGCGAGCTGGTGTCCCAGCAGAACTTTAGCGTGTGGGCCCTGAACCCCCAGACCTACAGACTGGAACGGGCCAGAGTGTCCCGGGCCTTCTGTACCGGAATCAAGCCCGTGTACCGGCTGACCACACGGCTGGGCAGATCCATCAGAGCCACCGCCAACCACCGGTTTCTGACACCCCAGGGCTGGAAGAGAGTGGACGAACTGCAGCCCGGCGACTACCTGGCCCTGCCCAGAAGAATCCCTACCGCCAGCGGCAGCGGCGGCGAGAACCTGTACTTCCAGGGCGAGAACCTGTACTTCCAGGGCGGCAGCGGCGGCGCCGCTGCCTGCCCTGAGCTGAGACAGCTGGCACAGAGCGACGTGTACTGGGACCCCATCGTGTCCATCGAGCCCGACGGCGTGGAAGAGGTGTTCGACCTGACAGTGCCTGGCCCCCACAACTTCGTGGCCAACGACGGATCCGCTCAGGTGCAGCTGGTGGAATCTGGTGGTAGACTGGTGCAGGCCGGCGATAGCCTGAGACTGTCTTGTGCCGCCAGCGGCAGAACCTTTAGCACATCTGCCATGGCCTGGTTCAGACAGGCCCCTGGCAGAGAAAGGGAATTCGTGGCCGCCATCACATGGACCGTGGGCAATACCATCCTGGGCGACTCTGTGAAGGGCAGATTCACCATCAGCCGGGACAGAGCCAAGAACACCGTGGACCTGCAGATGGACAACCTGGAACCTGAGGACACCGCCGTGTACTACTGCAGCGCCAGATCTAGAGGCTACGTGCTGTCCGTGCTGAGAAGCGTGGACAGCTACGATTATTGGGGCCAGGGCACCCAAGTGACCGTGTCTTGAATCTAGACCTTCTGCGGGGCTTGCCTTCTGGCCATGCCCTTCTTCTCTCCCTTGCACCTGTACCTCTTGGTCTTTGAATAAAGCCTGAGTAGGAAAAAAAAAAAAAAAAAAAAAAAAAAAAAAAAAAAAAAAAAAAAAAAAAAAAAAAAAAAAAAAAAAAAAAAAAAAAAAAAAAAAAAAAAAAAAAAAAAAAAAAAAAAAAAAAAAAAAAAA]

- **MS2CP(1-45)-RmaN(-4)^Scage^-Lag16**

Template pDNA: pUTR2-MS2CP(1-45)-RmaN(-4)^Scage^-Lag16

Primers: HNT-34 and HNT-9

T7 promoter (for CleanCap AG Reagent): 11-30, Kozak sequence (including start codon): 79-87, MS2CP N-terminal fragment gene: 88-222, Caged Rma N-intein gene: 223-684, anti-GFP nanobody Lag16 gene: 691-1071, Stop codon: 1072-1074

[CAGTGAATTGTAATACGACTCACTATAAGGCGAATTAAGAGAGAAAAGAAGAGTAAGAAGAAATATAAGACACCGGTCGCCACCATGGCTTCTAACTTTACTCAGTTCGTTCTCGTCGACAATGGCGGAACTGGCGACGTGACTGTCGCCCCAAGCAACTTCGCTAACGGGGTCGCTGAATGGATCAGCTCTAACTCGCGATCACAGGCTTACAAAGTAACCTGTCTGGCCGGCGATACCCTGATCACCCTGGCCGATGGCAGAAGAGTGCCCATCCGCGAGCTGGTGTCCCAGCAGAACTTTAGCGTGTGGGCCCTGAACCCCCAGACCTACAGACTGGAACGGGCCAGAGTGTCCCGGGCCTTCTGTACCGGAATCAAGCCCGTGTACCGGCTGACCACACGGCTGGGCAGATCCATCAGAGCCACCGCCAACCACCGGTTTCTGACACCCCAGGGCTGGAAGAGAGTGGACGAACTGCAGCCCGGCGACTACCTGGCCCTGCCCAGAAGAATCCCTACCGCCAGCGGCAGCGGCGGCGAGAACCTGTACTTCCAGGGCGAGAACCTGTACTTCCAGGGCGGCAGCGGCGGCGCCGCTGCCTGCCCTGAGCTGAGACAGCTGGCACAGAGCGACGTGTACTGGGACCCCATCGTGTCCATCGAGCCCGACGGCGTGGAAGAGGGATCCGCTCAGGTGCAGCTGGTGGAATCTGGTGGTAGACTGGTGCAGGCCGGCGATAGCCTGAGACTGTCTTGTGCCGCCAGCGGCAGAACCTTTAGCACATCTGCCATGGCCTGGTTCAGACAGGCCCCTGGCAGAGAAAGGGAATTCGTGGCCGCCATCACATGGACCGTGGGCAATACCATCCTGGGCGACTCTGTGAAGGGCAGATTCACCATCAGCCGGGACAGAGCCAAGAACACCGTGGACCTGCAGATGGACAACCTGGAACCTGAGGACACCGCCGTGTACTACTGCAGCGCCAGATCTAGAGGCTACGTGCTGTCCGTGCTGAGAAGCGTGGACAGCTACGATTATTGGGGCCAGGGCACCCAAGTGACCGTGTCTTGAATCTAGACCTTCTGCGGGGCTTGCCTTCTGGCCATGCCCTTCTTCTCTCCCTTGCACCTGTACCTCTTGGTCTTTGAATAAAGCCTGAGTAGGAAAAAAAAAAAAAAAAAAAAAAAAAAAAAAAAAAAAAAAAAAAAAAAAAAAAAAAAAAAAAAAAAAAAAAAAAAAAAAAAAAAAAAAAAAAAAAAAAAAAAAAAAAAAAAAAAAAAAAAA]

- **GFPenhNb-RmaC-MS2CP(46-116)-VPg(FCV)**

Template pDNA: pUTR2-GFPenhNb-RmaC-MS2CP(46-116)-VPg(FCV)

Primers: HNT-34 and HNT-9

T7 promoter (for CleanCap AG Reagent): 11-30, Kozak sequence (including start codon): 79-87, GFP enhancer nanobody gene: 88-432, Rma C-intein gene: 439-591, MS2CP C-terminal fragment gene: 592-804, FCV-derived VPg gene: 811-1143, Stop codon: 1144-1146

[CAGTGAATTGTAATACGACTCACTATAAGGCGAATTAAGAGAGAAAAGAAGAGTAAGAAGAAATATAAGACACCGGTCGCCACCATGGCTCAGGTGCAGCTTGTTGAATCTGGCGGAGCACTGGTTCAGCCTGGCGGATCTCTGAGACTGTCTTGTGCCGCCTCTGGCTTCCCCGTGAACCGGTACAGCATGCGGTGGTATAGACAGGCCCCTGGCAAAGAACGAGAGTGGGTGGCCGGAATGTCTAGCGCTGGCGATAGAAGCAGCTACGAGGACAGCGTGAAGGGCAGATTCACCATCAGCAGGGACGACGCCAGAAACACCGTGTACCTGCAGATGAACAGCCTGAAGCCTGAGGACACCGCCGTGTACTACTGCAACGTGAACGTGGGCTTCGAGTACTGGGGCCAGGGAACCCAAGTGACCGTTTCTGGATCCGCCGCTGCCTGCCCTGAGCTGAGACAGCTGGCACAGAGCGACGTGTACTGGGACCCCATCGTGTCCATCGAGCCCGACGGCGTGGAAGAGGTGTTCGACCTGACAGTGCCTGGCCCCCACAACTTCGTGGCCAACGACATCATTGCCCACAACTGTAGCGTTCGTCAGAGCTCTGCGCAGAATCGCAAATACACCATCAAAGTCGAGGTGCCTAAAGGCGCATGGAGGTCTTACTTAAATATGGAACTAACCATTCCAATTTTCGCCACGAATTCCGACTGCGAGCTTATTGTTAAGGCAATGCAAGGTCTCCTAAAAGATGGAAACCCGATTCCCTCGGCCATCGCGGCCAACTCCGGCATCTACGGATCCGCCAAGGGCAAGACCAAGAGCAAAGTGGGCCCCTACAGAGGCAGAGGCGTGGCCCTTACAGACGACGAGTATGACGAATGGCGCGAGCACAACGCCACCAGAAAGCTGGATCTGAGCGTGGAAGATTTCCTGATGCTGCGGCACAGAGCCGCTCTGGGAGCTGATGATGCCGACGCCGTGAAGTTCAGATCCTGGTGGAACAGCAGAAGCCGGCTGGCCGACGATTACGAGGATGTGACCGTGATCGGCAAAGGCGGCGTGAAGCACGAGAAGATCCGGACCAATACTCTGAGAGCCGTGGACAGAGGCTACGACGTGTCCTTCGCTGAAGAATGAATCTAGACCTTCTGCGGGGCTTGCCTTCTGGCCATGCCCTTCTTCTCTCCCTTGCACCTGTACCTCTTGGTCTTTGAATAAAGCCTGAGTAGGAAAAAAAAAAAAAAAAAAAAAAAAAAAAAAAAAAAAAAAAAAAAAAAAAAAAAAAAAAAAAAAAAAAAAAAAAAAAAAAAAAAAAAAAAAAAAAAAAAAAAAAAAAAAAAAAAAAAAAAA]

- **GFPenhNb-RmaC^cage^-MS2CP(46-116)-VPg(FCV)**

Template pDNA: pUTR2-GFPenhNb-RmaC^cage^-MS2CP(46-116)-VPg(FCV)

Primers: HNT-34 and HNT-9

T7 promoter (for CleanCap AG Reagent): 11-30, Kozak sequence (including start codon): 79-87, GFP enhancer nanobody gene: 88-432, Caged Rma C-intein gene: 439-813, MS2CP C-terminal fragment gene: 814-1026, FCV-derived VPg gene: 1033-1365, Stop codon: 1366-1368

[CAGTGAATTGTAATACGACTCACTATAAGGCGAATTAAGAGAGAAAAGAAGAGTAAGAAGAAATATAAGACACCGGTCGCCACCATGGCTCAGGTGCAGCTTGTTGAATCTGGCGGAGCACTGGTTCAGCCTGGCGGATCTCTGAGACTGTCTTGTGCCGCCTCTGGCTTCCCCGTGAACCGGTACAGCATGCGGTGGTATAGACAGGCCCCTGGCAAAGAACGAGAGTGGGTGGCCGGAATGTCTAGCGCTGGCGATAGAAGCAGCTACGAGGACAGCGTGAAGGGCAGATTCACCATCAGCAGGGACGACGCCAGAAACACCGTGTACCTGCAGATGAACAGCCTGAAGCCTGAGGACACCGCCGTGTACTACTGCAACGTGAACGTGGGCTTCGAGTACTGGGGCCAGGGAACCCAAGTGACCGTTTCTGGATCCGGAATCAAGCCCGTGTACCGGCTGACCACACGGCTGGGCAGATCCATCAGAGCCACCGCCAACCACCGGTTTCTGACACCCCAGGGCTGGAAGAGAGTGGACGAACTGCAGCCCGGCGACTACCTGGCCCTGCCCAGAAGAATCCCTACCGCCAGCGGCAGCGGCGGCGAGAACCTGTACTTCCAGGGCGAGAACCTGTACTTCCAGGGCGGCAGCGGCGGCGCCGCTGCCTGCCCTGAGCTGAGACAGCTGGCACAGAGCGACGTGTACTGGGACCCCATCGTGTCCATCGAGCCCGACGGCGTGGAAGAGGTGTTCGACCTGACAGTGCCTGGCCCCCACAACTTCGTGGCCAACGACATCATTGCCCACAACTGTAGCGTTCGTCAGAGCTCTGCGCAGAATCGCAAATACACCATCAAAGTCGAGGTGCCTAAAGGCGCATGGAGGTCTTACTTAAATATGGAACTAACCATTCCAATTTTCGCCACGAATTCCGACTGCGAGCTTATTGTTAAGGCAATGCAAGGTCTCCTAAAAGATGGAAACCCGATTCCCTCGGCCATCGCGGCCAACTCCGGCATCTACGGATCCGCCAAGGGCAAGACCAAGAGCAAAGTGGGCCCCTACAGAGGCAGAGGCGTGGCCCTTACAGACGACGAGTATGACGAATGGCGCGAGCACAACGCCACCAGAAAGCTGGATCTGAGCGTGGAAGATTTCCTGATGCTGCGGCACAGAGCCGCTCTGGGAGCTGATGATGCCGACGCCGTGAAGTTCAGATCCTGGTGGAACAGCAGAAGCCGGCTGGCCGACGATTACGAGGATGTGACCGTGATCGGCAAAGGCGGCGTGAAGCACGAGAAGATCCGGACCAATACTCTGAGAGCCGTGGACAGAGGCTACGACGTGTCCTTCGCTGAAGAATGAATCTAGACCTTCTGCGGGGCTTGCCTTCTGGCCATGCCCTTCTTCTCTCCCTTGCACCTGTACCTCTTGGTCTTTGAATAAAGCCTGAGTAGGAAAAAAAAAAAAAAAAAAAAAAAAAAAAAAAAAAAAAAAAAAAAAAAAAAAAAAAAAAAAAAAAAAAAAAAAAAAAAAAAAAAAAAAAAAAAAAAAAAAAAAAAAAAAAAAAAAAAAAAA]
